# Heavy Metals of Santiago Island (Cape Verde) Alluvial Deposits: Baseline Value Maps and Human Health Risk Assessment

**DOI:** 10.3390/ijerph16010002

**Published:** 2018-12-20

**Authors:** Marina M. S. Cabral Pinto, Eduardo A. Ferreira da Silva

**Affiliations:** Department of Geosciences, GeoBioTec Research Centre, University of Aveiro, 3810-193 Aveiro, Portugal; eafsilva@ua.pt

**Keywords:** potentially harmful elements, alluvial deposits, baseline values (BV), human health risk assessment, Santiago Island, Cape Verde

## Abstract

The chemical composition of surface geological materials may cause metabolic changes and promote endemic diseases (e.g., oncological, gastrointestinal, neurological or cardiovascular diseases). The results of a geochemical survey is presented following the guidelines proposed by the International Project IGCP 259 performed on the alluvium of Santiago Island (Cape Verde) and focused on public health issues. Geochemical mapping is the base knowledge needed to determine critical contents of potential toxic elements and the potentially harmful regions in the planet. This work presents maps of baseline values of potentially toxic elements (As, Cd, Co, Cr, Cu, Hg, Mn, Ni, Pb, V, and Zn) in Santiago alluvium and the assessment of their human health risks. According to the results the Cd, Co, Cr, Ni and V baseline values are above the Canadian guidelines for stream sediments (for any proposal use) and for soils (for agricultural and residential proposal uses) and also above the target values of Dutch guidelines. Hazard indexes (HI) were calculated for children and adults. For children (HI) are higher than 1 for Co, Cr and Mn, indicating potential non-carcinogenic risk. For the other elements and for adults there is no potential non-carcinogenic risk. Cancer risk was calculated for Cd, Cr and Ni exposures, for adults and children, and the results are only slightly higher than the carcinogenic target risk of 1 × 10^−6^ for adults exposed to Cr by inhalation. However, these results may be underestimated because alluvial contaminants may be indirectly ingested by groundwater and by crop and vegetables consumption.

## 1. Introduction

Geochemical surveys were developed in the last century [1,2,3,4,5,6] mainly as a means of geochemical prospecting of ore deposits [7,8]. Geochemical databases have been carried out in many regions [9,10,11,12,13], countries [7,14,15,16,17,18], multinational regions [19] and, more recently, continents [20,21,22], at various scales, ranging from high to very low density (e.g., >1 sample/km^2^ to 1 sample/10,000 km^2^, respectively). Geochemical surveys application has expanded to also encompass environmental monitoring, land-use decision support, natural resource management, and medical geology [7,23]. A diversity of sampling media have been targeted by geochemical surveys over time, which includes rock, sediment, soil, alluvium, ground water, surface water, dust, and vegetation [22]. In order to understand the transference, mobility, residence and biogeochemical processes of chemical elements few recent surveys have even targeted several media [24,25].

Geochemical surveys are especially important in developing countries like Cape Verde, where intervention limits for near-surface environment are not yet established. Considering this lack of information a high-density (aprox. 1/3 km^2^) geochemical survey in Santiago Island, Cape Verde archipelago, following the guidelines proposed by the International Geological Correlation Program (IGCP) Project 259 [1], was conducted in order to compile the first environmental geochemical atlas for that region. This paper presents the baseline value (BV) Santiago Island maps of some potentially toxic elements in alluvial deposits. Alluvial deposits are important natural resources, in particular in areas of limited rainfall when weathering profiles are very thin or non-existent, being the most adequate place for local agriculture. Knowledge of the geochemistry of near-surface environment is essential as it contributes to the improvement of the nutritional status of the population [26,27] and helps to understand the causes of some endemic diseases which can persist through out the life course due to heavy metal exposure, such as As, Cr, Al, Mn, Pb, Hg, Cu, Co, etc. Some elements can naturally accumulate on alluvial deposits in concentrations that are toxic to the plant, to the animal feeding on it and to humans. The dispersion of these elements in the environment is mainly carried out by water and air, which are vehicles linking the inorganic environment to life. The inhabitants of Santiago Island depend on groundwater for consumption and for agriculture and the flux water-vegetables-human also deserve evaluation, because endemic diseases can be controlled with proper measures, if its cause is well constrained. Some of these potentially toxic elements (PTEs) can lead to long-term carcinogenic and non-carcinogenic health risks and early environmental and occupational exposure to PTEs in different locales around the world has been related to oncological disease and mortality, neurological or cardiovascular diseases [28,29,30,31,32,33,34,35,36,37,38,39,40], etc. The widespread existence of PTEs has become one of the most serious environmental concerns around the globe and the development of efficient and selective chemical sensors to detect such potentially toxic elements have been a priority [41,42,43,44,45,46,47,48].

This study had the following objectives: (1) present a geochemistry survey of alluvial deposits for the studied metals and metalloid; (2) define the baseline values on Santiago Island; (3) evaluate the environmental risks; and (4) evaluate the human health risk, in areas naturally enriched with metals.

## 2. Settings of the Archipelago of Cape Verde and Santiago Island

**Figure 1:**
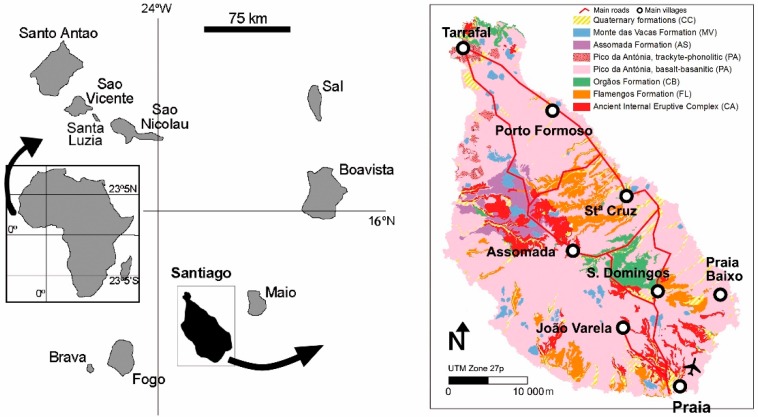
The Cape Verde Archipelago and its location on Senegal’s western coast, adapted from [7] and geological cartography of the island of Santiago, Cape Verde, adapted from [49].

The archipelago of Cape Verde (composed by 10 islands—Figure 1) is located at the eastern shore of the Atlantic Ocean, 500 km west from Senegal’s Cape Verde, off the western shore of Africa (Figure 1). Santiago island (991 km^2^), located in the southern part of the archipelago, is the biggest island and with half of the country´s population [7].

Santiago Island has a semi-arid climate, with strong winds during the dry season, and a mean annual precipitation of 321 mm, mainly due to torrential rains, during the wet season [50]. It has 215 km^2^ of arable area and estimated hydric resources of 56.6 × 106 m^3^/year on the surface, and 42.4 × 106 m^3^/year underground [51].

The works carried out by [49,52,53,54,55] allowed the establishment of the volcanostratigraphy of the island of Santiago (Figure 1). Table 1 provides a brief description of the geologic units in Santiago, named: Ancient Internal Eruptive Complex (CA), Flamengos Formation (FL), Orgãos Formation (CB), Eruptive Complex of Pico da Antónia (PA), Assomada Formation (AS), Monte das Vacas Formation (MV), and quaternary formations (CC).

## 3. Materials and Methods

### 3.1. Sample Collection, Chemical Analysis

Three hundred and forty near surface alluvium composite samples (0–15 cm depth) were sampled across the Santiago Island, at a density of approximately 0.3 site/km^2^. On each site the composite sample (~1 kg) was obtained by collecting five points, spaced approximately 50 m. These samples represent pristine alluvial deposits developed from all the geological formations in Santiago Island. To establish the sampling sites and the treatment of the samples, the guidelines of the IGCP 259 project were followed [1]. Field duplicate samples were taken at every 10 sites and locations affected by pollution were avoided. Samples were then sieved to <2 mm, being this fraction used for all chemical analyses. 

The chemical analysis was performed at ACME Analytical Laboratories, Ltd. (Vancouver, BC, Canada). Individual samples were digested in aqua regia and analysed by inductively coupled plasma-mass spectrometry (ICP-MS, ALS Global, Vancouver, Canada) for As, Cd, Co, Cr, Cu, Hg, Mn, Ni, Pb, V, and Zn. Digestion with aqua regia is a chemical attack method used in heavy metal environmental studies because it is effective in removing the more mobile elements normally associated with clay minerals, organic matter and other secondary minerals [1].

### 3.2. Analytical Quality Control, Statistical Analysis and Baseline Value

The data resulting from the chemical analysis of the elements was subjected to several data quality tests in order to determine which elements have reliable data to be interpreted by subsequent statistical analysis and is well described in Cabral Pinto et al. [7]. The methodology followed in this work to determine the baseline value of each metal at each sampling site followed the guidelines of the IGCP 259 project [1]. The mapping of the BVs was performed by ordinary kriging using a theoretical model of spatial continuity fitted to the experimental variograms calculated for each metal (Supplementary Materials, Table S1). Cross validation was carried out for each interpolated variable to assess if the fitted model was suitable for the experimental variogram. The root-mean-square error (RMSE) was used to measure the differences between values predicted by the model and the actual values. The RMSE ranges from 0 to infinity, with 0 corresponding to the ideal. The baseline value representative of the entire Santiago Island (BV-S) was calculated as the median of the data set limited by the Tukey range [56].

### 3.3. Risk Assessment

The environmental risk (ER) is calculated for PTE using Canadian [57] and Dutch [58] legislations for soils and sediments. For each element ER = BV-S/P, where computed. BV-S is the baseline element concentration in Santiago Island and P is the permissive level of that element, according to the legislations.

The non-carcinogenic and carcinogenic risks for residents of the Santiago Island were estimated according the United States Environmental Protection Agency (USEPA) methodology [59]. The equations used to determine the exposition to toxic elements via ingestion, dermal contact and inhalation are:

(1) $${\mathrm{CDI}}_{\mathrm{ingest}}=\frac{C\times \mathrm{IRS}\times \mathrm{EF}\times \mathrm{ED}}{\mathrm{BW}\times \mathrm{AT}}\times \mathrm{CF}$$

(2) $${\mathrm{CDI}}_{\mathrm{dermal}}=\frac{C\times \mathrm{SA}\times \mathrm{AF}\times \mathrm{ABS}\times \mathrm{EF}\times \mathrm{ED}}{\mathrm{BW}\times \mathrm{AT}}\times \mathrm{CF}$$

(3) $${\mathrm{CDI}}_{\mathrm{inhalation}}=\frac{C\times \mathrm{ET}\times \mathrm{EF}\times \mathrm{ED}}{\mathrm{PEF}\times 24\times \mathrm{AT}}$$

where CDI = Chronic Daily Intake (µg/kg bw/day); C—Concentration of chemical in alluvial deposits (mg kg^−1^); IRS—Ingestion Rate of Alluvium (mg day^−1^); EF—Exposure Frequency (350 days/year); ED—Exposure Duration (35 years for adult; 6 year for children); BW—Body Weight (70 kg for adult; 15 kg for children); AT—Averaging Time (365 days); CF—Conversing Factor (10^−6^ kg mg^−1^); SA—Skin Surface Area available for contact (2373 cm^2^ for children and 6032 cm^2^); AF—Alluvium to skin Adherence Factor (0.2 mg·cm^−2^ for children and 0.07 mg·cm^−2^ for adult); ABS—Absorption Factor (0.001); ET—Exposure Time (12 h·day^−1^); PEF—Particle Emission Factor: 1.36 × 109 m^3^·kg^−1^ [60]. In the formulas C is the concentration of PTE in alluvial deposits, which is the 95% upper confidence limit (UCL) in accordance with the USEPA [61].

The human health risk caused by PTEs exposure is expressed as hazard quotient (HQ) = ADD/RfD. The ADD is the average daily dose that a children or adult is exposed. RfD is the reference dose which is the daily dosage that enables the exposed individual to sustain this level. The HI is the chronic hazard index that is the sum of the hazard quotient for multiple exposure pathways. When HI values >1, there is a chance that non-carcinogenic risk may occur; otherwise, HI < I the opposite applies. The carcinogenic risks were calculated for Cd, Cr and Ni exposure of Santiago Island population, according to the Exposure Factors Handbook [60,62] and using the Slope Factors according to [63]. USEPA acceptable risk values for cancer risk 1 × 10^−4^ to 1 × 10^−6^.

## 4. Results and Discussion

### 4.1. Baseline Value Maps

**Figure 2:**
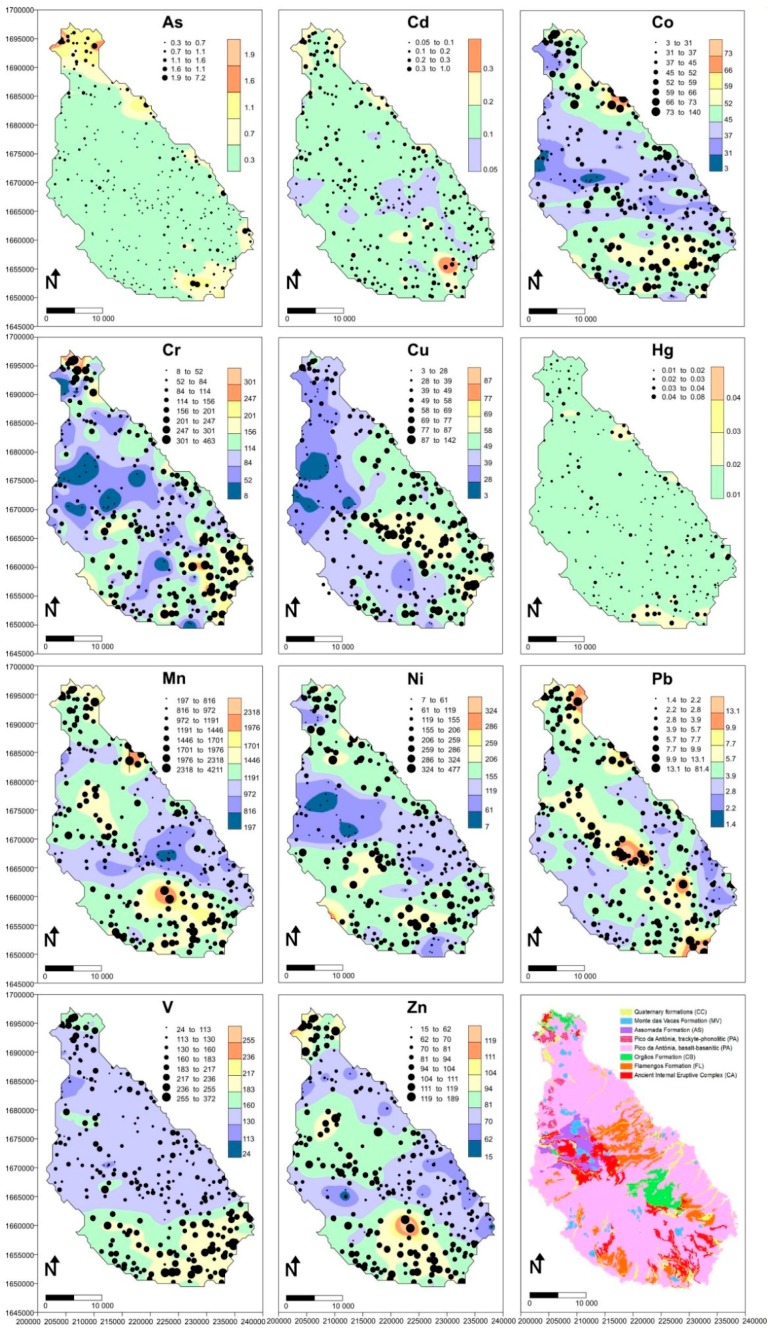
Spatial distribution of the As, Cd, Co, Cr, Cu, Hg, Mn, Ni, Pb, V and Zn baseline values. Coordinate system: UTM zone 27p. The geological map of Figure 1 is shown in the last panel for easier comparison with the BV spatial fields.

Descriptive statistics including the median, mean ± standard deviation, coefficient of variation percent, and the ranges of the analyzed metals concentrations along with the BV-S of Santiago Island were calculated and listed in Table 2. Figure 2 displays the spatial distribution of geochemical baseline values of the studied PTE at the sampling locations and also at all points of the interpolated spatial field.

The mineralogical composition of alluvial deposits is primarily governed by the mineralogy of the bedrock, climatic conditions (precipitation, temperature, wind direction), and topography. Chemical weathering is not intense in Santiago, due to the semiarid climatic conditions and the vigorous relief. The alluvium mineralogy is a combination of components inherited from the original lithology, minerals resulting from the alteration of these primary components, and probably also wind-transported minerals, mainly from the Sahara Desert. The alluvial deposits on the island of Santiago are dominated by primary silicate minerals such as pyroxene, feldspar and olivine (Table 1). The main secondary minerals are phyllosilicates (smectite, palygorskite, kaolinite, mica/illite), calcite, hematite and also quartz. Other minerals occur, such as leucite, analcite, apatite, nepheline, magnetite, titanomagnetite, ilmenite, chromite, garnet, serpentine, zeolites, siderite, opal, barite, titanite, zircon, halite, aragonite, dolomite, brucite, and chlorite [23]. A brief description of soils types of Santiago Island is present in the Supplementary Materials (Table S2).

Figure 2 and Table 1 and Table 3 shows that the alluvial deposits of Santiago Island, Cabo Verde, have a geochemical composition controlled by the type of underlying rock [23,64]. Most of the elements in the alluvium have a mainly geogenic origin.

### 4.2. Risk Assessment

The comparison of the BV-S of each studied PTE in the alluvial deposits of Santiago Island and the international values legislated as dangerous for certain uses (guideline values), is given in Table 3. The BV-S of Co, Cr, Cu, Ni and V are above the Canadian guidelines [57] for soils (for agricultural and residential uses), stream sediments (for any use) and above the target values of Dutch guidelines [58]. Manganese has not international guidelines for any property use, but its baseline value in Santiago alluvium is enriched relatively to upper crust values (774 mg kg^−1^) of Rudnick and Gao [65].

The Hazard Quotients (HQ) for ingestion, dermal contact and inhalation routes and Hazard Indexes (HI) were calculated (Table 4), for the PTE which are above the international guidelines for diverse property uses and also for Mn. Environmental exposure to Mn can induce parkinsonism and although the long-term medical significance of this finding is unclear, the data are troubling and point to the need for further investigation of manganese’s health risks [66]. The selected elements are potentially toxic elements and some (Cd, Cr and Ni) are also carcinogenic [67]. The pathways chosen were ingestion, inhalation and dermal contact and the calculations were performed for children and adults.

The non-carcinogenic hazard indexes (HI) for all nine elements are presented in Table 4. For adults the HI are always smaller than 1, whereas for children they are higher than 1 for Co (HI = 2.9), Cr (HI = 1.1), and Mn (HI = 1.1), indicating potential non-carcinogenic risk. The HI value of these elements is mainly controlled by the HQ ingestion, which are also higher than 1 for these 3 elements (Table 4 and Supplementary Figure S1). For the other elements and for adults there is no potential non-carcinogenic risk. The HI is Co > Cr > Mn, for both children and adults. Compared to adult, children health index is higher, and their cumulative effect is also of concern for children. The high concentrations of PTE on near-surface environment can threaten human health via ingestion by geophagism, rare in adults, but quite common in children or by hand-to-mouth intake [68,69], and by inhalation of dust particles and by dermal contact, specially by farmers and construction workers. Potentially toxic elements can also threaten human health indirectly also by ingestion of contaminated groundwater. The studies of Türkdoğan et al. [70] show that high contents of these metals in volcanic soils, vegetables and fruits are related with endemic upper gastrointestinal disease region of Turkey.

The evaluation of cancer risk was performed for heavy metals which are potentially carcinogenic [66]. The results for cancer risk are higher than the carcinogenic target risk of 1 × 10^−6^ [60] only for Cr, for adults (Table 5). However, these results may be miscalculating the risk because the other pathways were not considered and contaminants may be indirectly ingested by groundwater and by crop and vegetables consumption. Pallegriti et al. [71] found that residents of Catania province with its volcanic region appear to have a higher incidence of papillary thyroid cancer than elsewhere in Sicily. Hawaii and the Philippines have revealed an increased incidence of thyroid cancer in volcanic areas [72]. The common denominator of these regions is their numerous volcanoes and the fact that several constituents of volcanic lava have been postulated as being involved in the pathogenesis of thyroid cancer. High incidences of thyroid cancer were found in volcanic areas (Hawaii and Iceland) and Malandrino et al. [73] found conclude that a volcanic environment may play a role in the pathogenesis of thyroid cancer. The areas of high incidence of endemic Kaposi sarcoma are characterised by a common geologic substrate, composed of fertile reddish-brown volcanic clay soils [74]. Chronic exposure to Cr has long been recognized as being capable to increase thyroid, sarcoma, lung, and, head cancer incidence among exposed human populations [75,76,77,78,79].

## 5. Conclusions

Baseline maps show that the alluvial deposits of Santiago Island, Cabo Verde, have a geochemical composition controlled by the type of underlying rock, as most of the elements in the alluvium have mainly a geogenic origin. The environmental risk calculations shown that the Santiago Island alluvial deposits are naturally contaminated in Co, Cr, Cu, Ni and V, because these elements have contents well above those allowed by Canadian and Dutch legislations for soils, for agricultural and residential property uses (intervention limits for soils are not yet established in Cape Verde). The non-carcinogenic hazard indexes (HI) were calculated for eight potentially toxic elements and they are always smaller than 1 for adults, considering that the alluvium contaminants enter the human body by soil ingestion, dermal contact and inhalation of dust particles. Most significant, non-carcinogenic Co hazard is 2.9 for children. Ingestion mainly controls the HI values, and ingestion by geophagism and by hand-to-mouth intake is much more common in children. The cancer risk is always lower or very close to the carcinogenic target value. Exposure to other pollutants through other media, such as the vegetables and water ingestion may constitute another sink of risk, that should be study in the future. The inhabitants of Santiago Island depend on groundwater for consumption and for agriculture and the flux water-vegetables-human also deserve evaluation, because endemic diseases can be controlled with proper measures, if its cause is well constrained.

## Figures and Tables

**Table 1:** Geologic units, outcrop, rock type and composition, primary minerals of Santiago Island, Cape Verde.

Geological Formation	Outcrop	Rock Type	Composition	Primary Minerals
CA—Ancient internal eruptive complex	Centre, centre-W and in stream valleys	Subaerial and submarine lava flows and pyroclastic deposits; dykes and intrusive rocks	Basalts-basanites, phonolites-trachytes and carbonatites	Feldspar pyroxene carbonates, olivine, phyllosilicates
FL—Flamengos formation	NE-flank of the island	Submarine lava flows with subordinated breccias and tuffs	Basanites	Pyroxene, Fe-Ti oxides, olivine, feldspar
CB—Orgãos formation	Centre-E	Volcano-sedimentary deposits; rare lava flowss	Diverse	Pyroxene, Fe-Ti oxides, carbonates, feldspar
PA—Pico da Antónia eruptive complex	Widespread in the island	Subaerial and submarine lava flows, dykes and pyroclastic material; intercalated sedimentary deposits	Basalts-basanites, phonolites-trachytes and conglomerates	Pyroxene, Fe-Ti oxides, feldspar olivine
AS—Assomada formation	Centre-W	Subaerial lava flows and some pyroclasts	Basanites	Pyroxene, Fe-Ti oxides, feldspar, olivine
MV—Monte das Vacas formation	50 cinder cones throughout the island	Subaerial pyroclasts and small subordinated lava flows	Basanites	Pyroxene, Fe-Ti oxides, feldspar, olivine
CC—recent sedimentary formations	Mostly in stream valleys	Alluvial, aeolian and marine deposits	Diverse	Pyroxene, Fe-Ti oxides, carbonates, feldspar

**Table 2:** Statistical As, Cd, Co, Cr, Cu, Hg, Mn, Ni, Pb, V and Zn variables analysed, interval ranges, and the baseline values (BV-S) of metals from the alluvial deposits of Santiago Island (*n* = 340). Values expressed in mg kg^−1^.

Variable	Median	Mean	SD	CV	Range	P_5_–P_95_	Tukey Range	BV-S
As	0.3	0.6	0.6	1.07	0.3–7.2	0.3–1.6	0.3–1.4	0.25
Cd	0.10	0.14	0.09	0.64	0.05–1.00	0.05–0.30	0.05–0.35	0.10
Co	44.7	45.1	13.9	0.31	3.1–140	26.4–66.1	15.8–73.4	44.65
Cr	114.0	124	68	0.55	8.0–463	20.0–251.5	8.0–264.0	114
Cu	48.8	48.6	18	0.37	3.2–142	17.6–77.8	9.4–87.6	48.7
Hg	0.01	0.01	0.01	0.74	0.01–0.08	0.01–0.03	0.01–0.04	0.01
Mn	1191	1260	442	0.35	197–4210	737–1976	255–2162	1182
Ni	155	161	76	0.47	6.8–477	21.3–286	6.8–338	154
Pb	3.9	5.2	6.6	1.26	1.4–81.4	2.0–10.1	1.4–10.1	3.80
V	160	161	45.7	0.28	24.0–372	92.4–236	50.5–263	159
Zn	81.0	82.7	19.1	0.23	15.0–199	57.0–189	34.0–130	81

Med: median; SD: standard deviation; CV: variation coefficient; P_5_–P_95_: the interval limited by the 5th and 95th percentile values; Tukey range (92) or non-anomalous range: P_25_ − 1.5 × (P_75_ − P_25_) − P_75_ + 1.5 × (P_75_ − P_25_); BV-S (baseline value for Santiago): the median of the data limited by the Tukey range.

**Table 3:** Baseline values of PTE from the alluvial deposits of Santiago, and admissible levels (in mg kg^−1^) in soils and stream sediments according to the Ontario (88) and Dutch guidelines (89).

Element	BV-S	Canadian Guidelines			Dutch Guidelines
		Soil Agricultural Property Uses	Soil Residential Property Uses	Sediments (All Types of Property Uses	Target Values
As	0.25	11	18	6	29
Cd	0.1	1	1.2	0.6	0.8
Co	**44.7**	**19**	**21**	50	**9**
Cr	**114**	**67**	**70**	**26**	**100**
Cu	48.7	62	92	**16**	**36**
Hg	0.01	0.3	0.2	0.2	0.3
Mn	1182	-	-	-	-
Ni	**154**	**37**	**82**	**16**	**36**
Pb	3.8	45	129	31	85
V	**159**	**86**	**86**	**90**	-
Zn	81	290	290	120	140

Note: The bold values highlight the concentrations of PTE of guidelines which are below the respectively concentrations of baseline values in Santiago alluvium.

**Table 4:** HQ values for various pathways and elements and HI for elements from Santiago Island.

Element	HQ Ingestion		HQ Dermal		HQ Inhalation		HI	
	Children	Adult	Children	Adult	Children	Adult	Children	Adult
Co	2.9 × 10^0^	3.1 × 10^−1^	8.2 × 10^−3^	1.2 × 10^−3^	4.1 × 10^−3^	2.3 × 10^−3^	**2.9**	0.3
Cr	1.1 × 10^0^	1.2 × 10^−1^	3.1 × 10^−3^	4.7 × 10^−4^	9.2 × 10^−4^	5.2 × 10^−4^	**1.1**	0.1
V	1.9 × 10^−1^	2.0 × 10^−2^	5.3 × 10^−4^	8.1 × 10^−5^	1.2 × 10^−3^	6.7 × 10^−4^	0.2	0.0
Ni	2.6 × 10^−2^	2.7 × 10^−3^	7.2 × 10^−5^	1.1 × 10^−5^	7.2 × 10^−7^	4.0 × 10^−7^	0.0	0.0
Cu	6.2 × 10^−1^	6.7 × 10^−2^	1.7 × 10^−3^	2.7 × 10^−4^	8.8 × 10^−4^	5.0 × 10^−4^	0.6	0.1
Zn	8.4 × 10^−3^	9.0 × 10^−4^	2.3 × 10^−5^	3.6 × 10^−6^	2.3 × 10^−7^	1.3 × 10^−7^	0.0	0.0
Cd	4.0 × 10^−3^	4.3 × 10^−4^	4.5 × 10^−4^	6.8 × 10^−5^	1.1 × 10^−5^	6.3 × 10^−6^	0.0	0.0
Mn	1.1 × 10^0^	1.2 × 10^−1^	3.1 × 10^−3^	4.7 × 10^−4^	1.5 × 10^−2^	8.3 × 10^−3^	**1.1**	0.1

Note: Bold values show the PTE values above 1, and so highlight the elements which have potential non-carcinogenic risk.

**Table 5:** Cancer risk values for Cr, Ni and Cd from Santiago Island.

Element	Cancer risk	
	Children	Adult
Cr	3.2 × 10^−7^	**1.1 × 10^−6^**
Ni	7.7 × 10^−9^	2.5 × 10^−8^
Cd	5.8 × 10^−11^	1.9 × 10^−10^

Note: Bold values show the PTE values above 1, and so highlight the elements which have potential non-carcinogenic risk.

## Supplementary Materials

Supplementary Materials: The following are available online at http://www.mdpi.com/1660-4601/16/1/2/s1, Figure S1: Hazard index (HI) and hazard quotient (HQ) for potentially toxic elements from Santiago Island. Symbols: HI-crosses; HQ for ingestion-balls; HQ for dermal contact—squares; HQ for inhalation-lines., Table S1: Parameters of the theoretical models of spatial continuity fitted to the experimental variogram of As, Cd, Co, Cu, Cr, Hg, Mn, Ni, Pb, V and Zn.
