# Supplementary material for: Heavy Metals of Santiago Island (Cape Verde) Alluvial Deposits: Baseline Value Maps and Human Health Risk Assessment
Source: Int J Environ Res Public Health. 2018 Dec 20;16(1):2. doi: 10.3390/ijerph16010002 (PMC6338986; doi:10.3390/ijerph16010002)
Supplement: Supplementary file 1 [file ijerph-16-00002-s001.pdf]

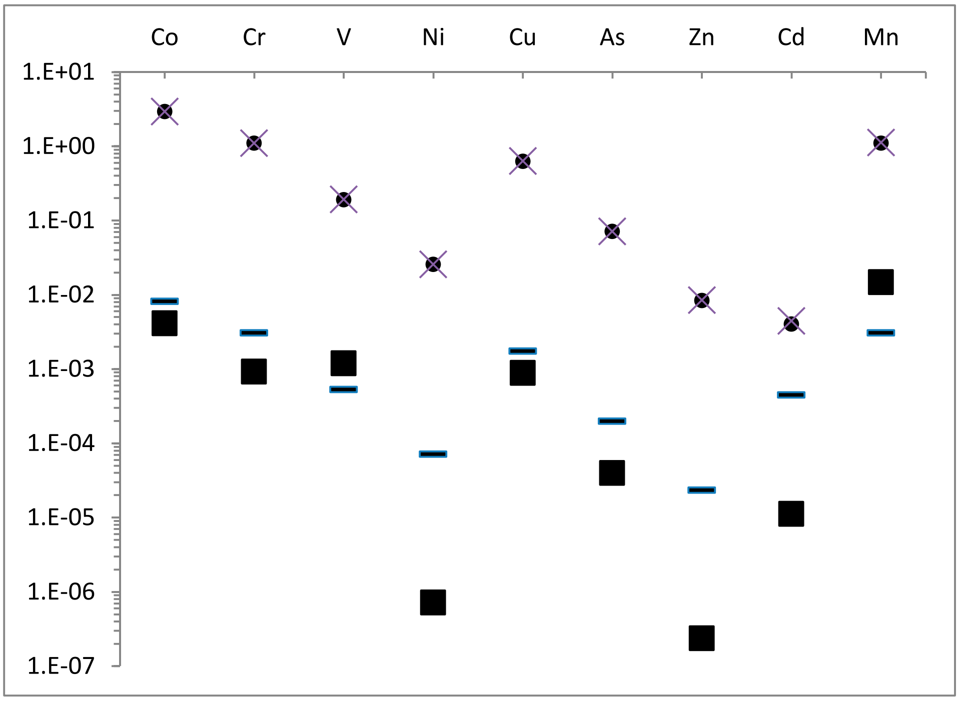

Figure S1. Hazard index (HI) and hazard quotient (HQ) for potentially toxic elements from Santiago Island. Symbols: HI-crosses; HQ for ingestion- balls; HQ for dermal contact – squares; HQ for inhalation- lines.

Table S1. Parameters of the theoretical models of spatial continuity fitted to the experimental variogram of As, Cd, Co, Cu, Cr, Hg, Mn, Ni, Pb, V, and Zn.

ID	Model	Main direction	C_0	C_1	Length	Anisotropy ratio	RMSE
As	exponential	90	0.1	0.19	8500	2.21	0.51
Cd	exponential	90	0.001	0.007	4000	1.45	0.16
Co	exponential	0	30	150	4000	1.27	2.38
Cr	exponential	45	2100	1900	2000	1.64	3.45
Cu	exponential	0	160	200	5000	1.22	1.05
Hg	spherical	0	0.0001	0.0005	6000	2.03	0.02
Mn	exponential	90	90000	140000	5000	1.17	5.03
Ni	exponential	30	2600	2800	3500	1.83	4.82
Pb	exponential	45	18	50	4000	2.73	4.80
V	exponential	0	1200	600	4000	1.98	3.56
Zn	spherical	75	150	200	4000	1.01	4.57

ID: Variable; Model: theoretical model fitted to the experimental variogram; C_0 – nugget effect; C_1 – sill for the structure; Length – major range in meters; Anisotropy ratio: Geometrical Anisotropy = major axis/minor axis; RMSE: root-mean-square error.
